# Adult essential extracorporeal membrane oxygenation (ECMO) skills for use in an e-learning program for ICU physicians, nurses and perfusionists: a consensus by a modified Delphi questionnaire

**DOI:** 10.1186/s12909-022-03764-2

**Published:** 2022-11-14

**Authors:** Harlinde Peperstraete, Annelien Steenhout, Filip De Somer, Pieter Depuydt, Eric Hoste, Isabelle Van Herzeele

**Affiliations:** 1 Department of Intensive Care Medicine, Ghent University Hospital, Ghent University, Ghent, Belgium; 2 Department of Anesthesiology, Ghent University Hospital, Ghent University, Ghent, Belgium; 3 Department of Cardiac Surgery, Ghent University Hospital, Ghent University, Ghent, Belgium; 4 Research Foundation-Flanders (FWO), Brussels, Belgium; 5 Department of Thoracic and Vascular Surgery, Ghent University Hospital, Ghent University, Ghent, Belgium

**Keywords:** Extra corporeal membrane oxygenation, Education, Modified Delphi, Consensus, ICU, Knowledge, Technical skill, Attitude

## Abstract

**Background:**

Education in ECMO starts with basic theory and physiology. For this type of training, self-assessment e-learning modules may be beneficial. The aim of this study was to generate consensus on essential ECMO skills involving various professional groups involved in caring for ECMO patients. These skills can be used for educational purposes: development of an e-learning program and fine-tuning of ECMO-simulation programs.

**Methods:**

Experts worldwide received an e-mail inviting them to participate in the modified Delphi questionnaire. A mixture of ECMO experts was contacted. The expert list was formed based on their scientific track record mainly in adult ECMO (research, publications, and invited presentations). This survey consisted of carefully designed questionnaires, organized into three categories, namely knowledge skills, technical skills, and attitudes. Each statement considered a skill and was rated on a 5-point Likert-scale and qualitative comments were made if needed. Based on the summarized information and feedback, the next round Delphi questionnaire was developed. A statement was considered as a key competency when at least 80% of the experts agreed or strongly agreed (rating 4/5 and 5/5) with the statement. Cronbach’s Alpha score tested internal consistency. Intraclass correlation coefficient was used as reliability index for interrater consistency and agreement.

**Results:**

Consensus was achieved in two rounds. Response rate in the first round was 45.3% (48/106) and 60.4% (29/48) completed the second round. Experts had respectively for the first and second round: a mean age of 43.7 years (8.2) and 43.4 (8.8), a median level of experience of 11.0 years [7.0-15.0] and 12.0 years [8.3-14.8]. Consensus was achieved with 29 experts from Australia (2), Belgium (16), France (1), Germany (1), Italy (1), Russia (2), Spain (1), Sweden, (1), The Netherlands (4). The consensus achieved in the first round was 90.9% for the statements about knowledge, 54.5% about technical skills and 75.0% about attitudes. Consensus increased in the second round: 94.6% about knowledge skills, 90.9% about technical skills and 75.0% about attitudes.

**Conclusion:**

An expert consensus was accomplished about the content of “adult essential ECMO skills”. This consensus was mainly created with participation of physicians, as the response rate for nurses and perfusion decreased in the second round.

**Supplementary Information:**

The online version contains supplementary material available at 10.1186/s12909-022-03764-2.

## Background

Extra Corporeal Membrane Oxygenation (ECMO) is increasingly used in modern Intensive Care Units (ICU’s), especially since the publication of the ECMO to rescue Lung injury in severe ARDS (EOLIA) trial, and H4N1 Influenza A and Severe Acute Respiratory Syndrome Coronavirus 2 (SARS-CoV-2) pandemics.[1–5] As this support becomes more widespread, there is also a growing need for proper training of physicians, perfusionists and ECMO nurses.[6–8] Taking care of ECMO-patients is not only technically demanding, but requires knowledge of certain pathophysiology. To master adequate problem solving in case of emergencies, different educational training programs with certification are organized worldwide. [9–12]

ELSO lists in its guideline all topics which an “ECMO-specialist”-program should cover, including theoretical concepts and hands-on water-drills. To start with, each participant must master the physiology and circuit components. The program needs to provide technical aspects like cannulation guidance, circuit changes and decannulation strategies. Cooperation from the different stake holders is trained with special attention to daily management, human factors and ethical considerations [8] These training sessions may result in a team whose ultimate goal is to discharge the patient with optimal quality of life. As indicated by the ELSO guideline, each ECMO center should develop center specific guidelines and policies for training ECMO specialists. Since these guidelines were published in February 2010, it is reasonable to suppose that they need updating. Teaching ECMO nurses, specialty registrars, clinical fellows and perfusionists and updating members of staff is time consuming not only for the tutors but also for the learners. In order to improve the accessibility and flexibility, developing an E-learning module can be the strategy to opt for.[11, 13] The aim of this study was to generate consensus on essential ECMO skills for developing an e-learning program and fine-tuning of the existing ECMO-simulation program at Ghent University Hospital, Belgium. A modified Delphi questionnaire approach was used.

## Methods

### Study design

This is a prospective international study using the modified Delphi methodology. The Delphi method was developed in the 1950s as a part of a US military defense project.[14] The modified Delphi technique is a structured consensus method, used in medical literature to accomplish an overall agreement or expert opinion about definitions, problems or other ideas. This method ensures that each participant can make judgments in complete anonymity, on an equal footing with the other participants, and can change his or her mind during the process. Experts are asked to give their opinion on statements in successive rounds. The evaluation is done by using an ordinal scale (e.g., Likert scale) for each statement, next to qualitative commentary in a text field. Until a certain level of consensus is reached, the procedure can be repeated. [14–16] In this study we used a five-point Likert scale. Consensus about a key competency in this study was defined by 80% of experts who rated a skill with a score of 4 or more and when the internal consistency shows a Cronbach Alpha score > 80%. This study aimed to accomplish consensus in two or three rounds.

### Experts

An expert was defined as an experienced health care professional (HCP), working in a high volume ECMO center. The high volume was estimated to be at least 20 cases a year. The physicians were active in intensive care unit (ICU), anesthesia, cardiac surgery, cardiology, and internal medicine. ICU nurses and perfusionists have also been interrogated. At the start of this survey 106 experts were contacted by e-mail. A mixture of ECMO experts was contacted. The expert list was formed based on their scientific track record mainly in adult ECMO (research, publications, and invited presentations).

### Questionnaires

Panel members were contacted by e-mail with information about the study and the purpose of the questionnaire. They were invited by an auto-generated e-mail to participate in the survey, which was created in the web-based software platform Research Electronic Data Capture (RedCap®). The research group consisted of two physicians (HP, IVH) and one perfusionist (FDS), experienced in ECMO and/or educational research from our center. This research group selected a list of statements based on several sources: “Extracorporeal Life Support: The ELSO Red Book (5th Edition), the “ECMO specialist training manual (4th Edition), the handbook of the course “ECMO-course for physicians and nurses” of the Leiden University Medical Center and the educational program from the Ghent University Hospital. The competency areas were grouped into three categories, namely knowledge skills, technical skills, and attitude. In the first round, experts were asked to score statements covering knowledge skills, technical skills, and attitude. Each skill was rated on a 5-point Likert-scale from ‘1, Strongly disagree’ to ‘5, Strongly agree’. For each topic, the panelist could make a proposal to change or nuance the statement. Pilot testing was done by a small group of individuals, who did not participate in the survey. After the first round, results were anonymously analyzed, and the suggested adjustments were applied. Based on the information obtained from round one, new statements and modifications proposed by panelists were collected and discussed in the research group to rephrase for clarity. The distributions of scores (median and interquartile range) for each statement from the first round were included within the second-round questionnaire. The experts were instructed in the second round to re-consider the statements presented in the first round, or slightly different statements and the newly added topics. In addition, a mock question was included. A mock question is inserted in a survey to check whether the participant is still focused, the answer is obvious, it ensures that the survey was not randomly completed.

### Data collection and data analysis

The completed surveys were collected by the software platform RedCap®. Information on demographics and expertise was collected through a questionnaire in the first round of the survey. Panelist were given six weeks to complete the study, with reminders sent out every week to non-responders. The second-round questionnaire was only sent out to experts who had completed the first round. Normal distribution of the responses was checked with the Shapiro Wilk test. Normal distributed variables are reported as mean (standard deviation), and non-normal distributed variables as median [interquartile range]. To determine the ranking of topics and skills from the questionnaire, median values of the panelists’ scores were used. The Cronbach’s Alpha score, for which an alpha value of 0.80 was chosen as an indicator of consensus, was calculated to test internal consistency.[17] The intraclass correlation coefficient (ICC) was used as the reliability index for interrater consistency and agreement. [18] To compare differences between educational backgrounds, the Kruskal-Wallis test was used, given the multiple testing, correction was made using the Bonferroni method. Wilcoxon-signed ranks test was used to compare ratings of the elements between the first and the second round. A p-value of 0.05 is determined as statistically significant. Statistical analysis was performed using SPSS 26.0 (Statistical Package for the Social Sciences, IBM Company, US).

### Ethics

This is a prospective, observational study in which the International Council for Harmonization (ICH) Good Clinical Practice (GCP) guideline is followed. Approval by the local research medical Ethics Committee of Ghent University Hospital was obtained on 30/07/2020 (reference number BC-07929). All participants had to give written consent prior to the start of the study and anonymity has been guaranteed.

## Results

### Demography

Of the 106 invited panelists, 54 started the survey, and 48 (45,3%) completed the first round. Fourteen of them were female (29.2%). The participants had a mean age of 43.7 (8.2) years with median experience of 11.0 [7.0-15.0] years. They worked in a unit with a median number of 45.0 [25.0-64.5] ICU beds and 94% of them worked in academic setting. The panel consisted of four (8.3%) nurses, 12 (25.0%) perfusionists and 32 (66.7%) physicians. 50% of them practiced in Belgium. In the second round the response rate was 29/48 (60.4%); 21 (72%) physicians, three (10%) nurses and five (17%) perfusionists completed the second round. Consensus was achieved with 29 experts from Australia (2), Belgium (16), France (1), Germany (1), Italy (1), Russia (2), Spain (1), Sweden, (1), The Netherlands (4).” (See Tables 1 and 2).

**Table 1:** Demographics and working experience

	Round 1	Round 2
Mean (SD) age	43.7 (8.2)	43.4 (8.8)
Median [IQR] years of experience with ECMO-patients	11.0 [7.0–15.0]	12.0 [8.3–14.8]
Median [IQR] number of ICU beds	45.0 [25.0-64.5]	45.0 [27.0–60.0]
Gender M/F, n (%)	34/14 (70.8/29.2)	21/8 (72.4/27.6)

**Table 2:** Countries where panelist were practicing

	Physicians		Nurses		Perfusionists		Total	
	Round 1	Round 2	Round 1	Round 2	Round 1	Round 2	Round 1	Round 2
	n	n	n	n	n	n	n	n
Country								
Australia	1	1			1	1	2	2
Belgium	15	11	3	2	6	3	24	16
France	1	1			1		2	1
Germany	1	1					1	1
Italy	2	1					2	1
Portugal					1		1	
Russia	3	2			1		4	2
Spain	2	1					2	1
Sweden	1	1					1	1
Switzerland	1				1		2	
The Netherlands	3	2	1	1	1	1	5	4
Turkey	1						1	
United Kingdom	1						1	
Total	32	21	4	3	12	5	48	29

### Delphi results

In the first round, experts were asked to score 56 statements covering 33 knowledge skills (including one mock question), 11 technical skills, and 12 attitudes. After analysis of this first round, four knowledge statements were added, and 46 statements were reformulated. Of the knowledge skills 28 (84.8%) skills were rephrased, all (100%) statements about technical skills were adapted, and seven (58.3%) attitude statements were changed. The changes ranged from minimal additions such as specifying who within the team should master the skill and clarifications of the statement. Consensus was accomplished in the first round in 45/56 (80,4%) of the statements, in the second round in 54/60 (90%) of the statements. There was excellent reliability when calculating Cronbach’s alfa, for the first round it was 0.921 and the second had 0.907. Regarding the first round, there was a significant interrater agreement between all 48 experts (ICC 0.861 with p < 0.001). The same was found in the second round with a significant interrater agreement between all 29 experts (ICC 0.942 with p < 0.001). For eight statements we found a significant difference in ratings between the first and second Delphi questionnaire, they were adjusted in between rounds. (Supplementary material)

### Knowledge

For the knowledge topics, in the first-round consensus was achieved in 90.9% of the topics and for 94.4% of the topics in the second round. Cronbach’s alfa was 0.909 for the first round and 0.864 for the second round. Two of the 36 knowledge topics could not be retained as key competencies and were removed from the list: “Pre-ECMO evaluation including RESP-score and SAVE-score (RESP = Respiratory Extracorporeal membrane Oxygenation Survival Prediction, SAVE = Survival After VA ECMO).” and “Knowledge of correct ECMO nomenclature.”

Also, the mock question was deleted. The three most important skills for knowledge were ranked as follows: (1) “Knowledge of ECMO physiology: in VA-ECMO: Optimization of hemodynamic support, including blood flow, native cardiac function.”, (2) “Knowledge of ECMO physiology: Optimization of pCO2.” and (3) “Knowledge of symptoms and clinical signs of limb ischemia and prevention of it.”. See Table 3.

**Table 3:** Top 3 rated skills for each of three categories and the skills without consensus

		Round 1							Round 2					
**KNOWLEDGE**		**Mean (SD)**			**Median** **[IQR]**			**Consensus %**	**Mean (SD)**			**Median [IQR]**		**Consensus %**
CONSENSUS														
Round 1: Knowledge of ECMO physiology: in VA-ECMO: Optimization of hemodynamic support Round 2: Knowledge of ECMO physiology: in VA-ECMO: Optimization of hemodynamic support, including blood flow and native cardiac function		4.65 (0.7)			5.0 [4.0–5.0]			96.55	4.79 (0.41)			5.0 [5.0–5.0]		100
Round 1 & 2: Knowledge of ECMO physiology: Optimization of pCO2.		4.56 (0.62)			5.0 [4.0–5.0]			89.66	4.75 (0.44)			5.0 [4.25-5.0]		100
Round 1 & 2: Knowledge of symptoms and clinical signs of limb ischemia and prevention of it.		4.73 (0.45)			5.0 [4.0–5.0]			100	4.72 (0.53)			5.0 [4.0–5.0]		96.55
NO CONSENSUS														
Round 1: Knowledge of pre-ECMO evaluation including RESP-score and SAVE-score (RESP = Respiratory Extracorporeal membrane Oxygenation Survival Prediction, SAVE = Survival After V-A ECMO). Round 2: For the physician, knowledge of pre-ECMO evaluation including RESP-score and SAVE-score (RESP = Respiratory Extracorporeal membrane Oxygenation Survival Prediction, SAVE = Survival After V-A ECMO).		3.80 (0.69)			4.0 [4.0–4.0]			77.1	3.95 (0.75)			4.0 [4.0–4.0]		79.3
Round 1: Knowledge of the principles of ECMO nomenclature. Round 2: Knowledge of correct ECMO nomenclature.		4.00 (0.72)			5.0 [3.5-5.0]			74.5	3.90 (0.64)			4.0 [3.25-4.0]		71.4
	**Round 1**									**Round 2**				
**TECHNICAL SKILLS**	**Mean (SD)**		**Median** **[IQR]**			**Consensus %**				**Mean (SD)**	**Median [IQR]**		**Consensus %**	
CONSENSUS														
Round 1: Being able to change the oxygenator Round 2: For perfusionist: being able to change the circuit / oxygenator.	3.80 (1.22)		4.0 [3.0–5.0]			66.67				4.76 (0.44)	5.0 [4.5-5.0]		100	
Round 1: Being able to prime the circuit Round 2: For the perfusionist: being able to prime the circuit.	3.66 (1.1)		4.0 [3.0–5.0]			60.71				4.72 (0.45)	5.0 [4.0–5.0]		100	
Round 1: Being able to insert / connect the cannulas correct. Round 2: For the physician and perfusionist: being able to insert / connect the cannulas correct.	4.28 (0.93)		5.0 [4.0–5.0]			82.14				4.61 (0.69)	5.0 [4.0–5.0]		96.43	
NO CONSENSUS														
Round 1: For the physician: being able to measure the vessel diameter and perform ultrasound guided puncture in peripheral ECMO. Round 2: For the physician: being able to measure the vessel diameter and perform ultrasound guided puncture in peripheral ECMO.	3.70 (1.08)		4.0 [3.0–5.0]			57.4				4.0 (0.97)	4.0 [3.0–5.0]		72.4	
	**Round 1**									**Round 2**				
**ATTITUDE**	**Mean (SD)**			**Median** **[IQR]**			**Consensus %**			**Mean (SD)**	**Median [IQR]**		**Consensus %**	
CONSENSUS														
Round 1 & 2: Knowledge of his/her limits and call for help if needed.	4.79 (0.46)			5.0 [5.0–5.0]			96.43			4.82 (0.35)	5.0 [5.0–5.0]		100	
Round 1 & 2: There should be an experienced team available 24/24 7/7 for troubleshooting.	4.77 (0.42)			5.0 [5.0–5.0]			100			4.76 (0.51)	5.0 [5.0–5.0]		96.55	
Round 1 & 2: Be able to consider the risks/ benefits for every ECMO run.	4.54 (0.54)			5.0 [4.0–5.0]			100			4.62 (0.49)	5.0 [4.0–5.0]		100	
NO CONSENSUS														
Round 1: To obtain an informed consent from the patient or family. Round 2: The physician should obtain an informed consent from the patient or family.	3.95 (0.88)			4.0 [3.0-4.5]			63.0			3.80 (0.89)	4.0 [3.0–4.0]		63.0	
Round 1 & 2: Handovers and communication should be structured and standardized e.g., using ISBAR.	4.15 (081)			4.0 [3.25-5]			73.3			3.95 (0.60)	4.0 [4.0–4.0]		75.9	
Round 1: Being an ELSO-member (Extracorporeal Life Support Organization) Round 2: The center should be registered as an ELSO-member (Extracorporeal Life Support Organization)	3.80 (0.89)			4.0 [3.0–5.0]			70.2			4.05 (0.68)	4.0 [4.0-4.75]		75.9	

Consensus in this study was defined by 80% of experts that rates a skill with a score of 4 or more

### Technical skills

Cronbach’s alfa was calculated 0.809 and 0.707, respectively for the first and the second round. In the first round there was only agreement for 54.5% of the skills, all statements have been reformulated. In the second-round consensus was achieved in 90.9% of the skills. This resulted in the removal of one technical skill: “For the physician: be able to measure the vessel diameter and perform ultrasound guided puncture in peripheral ECMO.” from the list, see also Table 3. The top three technical skills are: (1) “For the perfusionist: being able to change the circuit/oxygenator.”, (2) “For the perfusionist: priming of the circuit.”, (3) “For the physician and perfusionist: correct insertion/connection of the cannulas.”

### Attitude

In the category attitude, experts agreed in 75% of the statements in the first round and second round. Three of the 12 statements (25%) were not considered to be key competencies and were discarded, see Table 3. Calculated Cronbach’s alfa for attitude topics. was 0.714 and 0.778, respectively for the first and second round. The following statements made the top three: (1) “Know his/her limits and call for help if needed.”, (2) “There should be an experienced team available 24/24 7/7 for trouble-shooting.” and (3) “Be able to consider the risks/ benefits for every ECMO run.”.

Differences between professional groups for ranking a skill as an “essential skill”, were noted in 12 statements, and only in round one (Table 4).

**Table 4:** Skills rated differently between the three participating professional groups

Skill	Nurse score Median [IQR]	Perfusion score Median [IQR]	Physician score Median [IQR]	Consensus (%)	p
Knowledge					
R1: K1 Knowledge of the relevant vascular anatomy. R2: K1a Every team member should have the basic knowledge of the ‘classic’ vascular anatomy. R2: K1b Every physician should have knowledge of the echographic vascular anatomy.	2.5 [4.0–4.0] 4.0 [4.0-4.5] 4.0 [4.0-4.5]	4.0 [5.0–5.0] 4.0 [4.0–5.0] 4.0 [4.0–5.0]	4.0 [5.0–5.0] 5.0 [4.0–5.0] 4.0 [4.0–5.0]	93.8 93.1 93.1	0.0015^*^
R1: K3: Knowledge of the indications for V-A ECMO. R2: K3: Knowledge of the indications for V-A ECMO.	4.0 [4.0–4.0] 4.0 [4.0–4.0]	4.0 [4.0–5.0] 4.0 [4.0–5.0]	4.0 [4.0–5.0] 5.0 [5.0–5.0]	100 96.6	0.004^*^
R1: K4 Knowledge of the indications for V-V ECMO. R2: K4 Knowledge of the indications for V-V ECMO.	4.0 [4.0–4.0] 4.0 [4.0–4.0]	5.0 [4.0–5.0] 4.0 [4.0–5.0]	5.0 [4.0–5.0] 5.0 [5.0–5.0]	100 96.6	0.003^*^
R1: K5 Knowledge of the indications for eCPR (Extracorporeal CardioPulmonary Resuscitation). R2: K5 Knowledge of the indications - according to the local protocol - for eCPR (Extracorporeal cardiopulmonary resuscitation).	4.0 [4.0–4.0] 4.0 [3.5-4.0]	5.0 [5.0–5.0] 4.0 [4.0–5.0]	5.0 [4.0–5.0] 5.0 [4.0–5.0]	95.8 93.1	0.014^#^
R1: K8 Knowledge of the contraindications. R2: K8 Knowledge of the contraindications.	4.0 [4.0-4.5] 5.0 [4.5-5.0]	5.0 [4.0–5.0] 5.0 [4.0–5.0]	5.0 [4.5-5.0] 5.0 [4.5-5.0]	97.9 93.1	0.043^*^
R1: K21 Knowledge of prevention, diagnosis and treatment of Harlequin syndrome. R2: K21 Knowledge of prevention, diagnosis and treatment of Harlequin syndrome.	4.0 [4.0-4.5] 5.0 [4.5-5.0]	5.0 [5.0–5.0] 5.0 [5.0;5.0]	5.0 [4.0–5.0] 5.0 [4.0–5.0]	100 100	0.036^#^
R1: K32 Knowledge of the principles of ECMO nomenclature. R2: K32 Knowledge of correct ECMO nomenclature.	4.0 [3.5-4.0] 4.0 [3.5-4.0]	5.0 [4.0–5.0] 4.0 [4.0–4.0]	4.0 [3.5–4.5] 4.0 [3.5–4.5]	74.1 71.4	0.038^§^
Technical skills					
R1: T4 Being able to insert the guidewire correct and give attention for any signs of obstruction. R2: T4 For the physician: being able to insert the guidewire correct and give attention for any signs of obstruction.	3.0 [3.0-3.5] 5.0 [4.0–5.0]	5.0 [4.0–5.0] 5.0 [5.0–5.0]	5.0 [4.0–5.0] 5.0 [4.0–5.0]	73.9 93.1	0.033^*^
R1: T6 Being able to prime the circuit. R2: T6 For the perfusionists: being able to prime of the circuit.	3.0 [2.5-3.0] 5.0 [4.5-5.0]	4.0 [4.0–4.0] 5.0 [5.0–5.0]	4.0 [3.0–4.0] 5.0 [4.5-5.0]	93.6 100.0	0.041^#^
R1: T10 Being able to change the oxygenator. R2: T10 For perfusionists: being able to change the circuit / oxygenator.	2.0 [2.0-2.5] 5.0 [4.5-5.0]	4.0 [2.0–4.0] 5.0 [5.0–5.0]	5.0 [4.0–5.0] 5.0 [4.5-5.0]	64.4 100.0	0.039^*^
R1: T11 For physicians: being able to place an Avalon® cannula. R2: T11 Placement of a dual lumen canula should only be placed after multidisciplinary discussion by an experienced physician.	4.0 [4.0–4.0] 4.0 [3.5-4.0]	4.0 [4.0–5.0] 5.0 [5.0–5.0]	4.0 [3.0-4.5] 5.0 [4.0;5.0]	57.8 86.2	0.035^§^
Attitude					
R1: A3 To obtain an informed consent from the patient or family. R2: A3 The physician should obtain an informed consent from the patient or family.	2.0 [2.0-2.5] 4.0 [3.5-4.0]	4.0 [4.0–5.0] 4.0 [4.0–4.0]	4.0 [3.5-5.0] 4.0 [3.0–5.0]	63 65.5	0.025^*^

R1: Round 1 defined skill

R2: Round 2 defined skill

K: knowledge question, T: technical skill statement, A: attitude statement

* The p-values were calculated statistically significant different between nurses and physicians in R1

# The p-values were calculated statistically significant different between nurses and perfusionists in R1

§ The p-values were calculated statistically significant different between perfusionists and physicians in R1

A list of all the statements of the consensus can be found in Table 5.

**Table 5:** Statements of the first round (R1) and second round (R2) distribution parameters and consensus percentage

Statements of the first round (R1) and second round (R2) distribution parameters and consensus percentage	Mean (SD)		Median [IQR]		Consensus %
***Knowledge***					
R1: K1 Knowledge of the relevant vascular anatomy. R2: K1a Every team member should have the basic knowledge of the 'classic' vascular anatomy. R2: K1b Every physician should have knowledge of the echographic vascular anatomy.	4.45 (0.94) 4.50 (0.51) 4.35 (0.58)		5 [4–5] 4.50 [4–5] 4 [4–5]		93.8 93.1 93.1
R1: K2 Knowledge of the components of the ECMO circuit: drainage cannula, centrifugal pump, oxygenator, heating element, return cannula, gas blender, flow sensor. R2: K2 Every team member should be familiar with the components and monitoring of the ECMO circuit.	4.50 (0.51) 4.55 (0.60)		5 [4–5] 5 [4–5]		100 96.6
R1: K3 Knowledge of the indications for V-A ECMO. R2: K3 Knowledge of the indications for V-A ECMO.	4.50 (0.51) 4.60 (0.50)		5 [4–5] 5 [4–5]		100 96.6
R1: K4 Knowledge of the indications for V-V ECMO. R2: K4 Knowledge of the indications for V-V ECMO.	4.50 (0.51) 4.60 (0.50)		5 [4–5] 5 [4–5]		100 96.6
R1: K5 Knowledge of the indications for ECPR (Extracorporeal CardioPulmonary Resuscitation) ECMO. R2: K5 Knowledge of the indications - according to the local protocol - for eCPR (Extracorporeal cardiopulmonary resuscitation) ECMO.	4.55 (0.60) 4.50 (0.51)		5 [4–5] 4.50 [4–5]		95.8 93.1
R1: K6 Knowledge of when 'to convert' to another ECMO construction. R2: K6a For the physician and perfusion: knowledge of when to change the ECMO configuration. R2: K6b For the physician and perfusionists: knowledge of how to change the ECMO configuration.	4.40 (0.50) 4.70 (0.47) 4.55 (0.51)		5 [4–5] 5 [4–5] 5 [4–5]		89.4 100.0 100.00
R1: K7 Pre-ECMO evaluation including RESP-score and SAVE-score (RESP = Respiratory Extracorporeal membrane Oxygenation Survival Prediction, SAVE = Survival After V-A ECMO). R2: K7 For the physician: pre-ECMO evaluation including RESP-score and SAVE-score (RESP = Respiratory Extracorporeal membrane Oxygenation Survival Prediction, SAVE = Survival After V-A ECMO).	3.80 (0.69) 3.95 (0.75)		4 [4–4] 4 [4–4]		77.1 79.3
R1: K8 Knowledge of the contraindications. R2: K8 Knowledge of the contraindications.	4.65 (0.58) 4.70 (0.47)		5 [5–5] 5 [4–5]		97.9 93.1
R1: K9 Knowledge of ECMO physiology: Optimization of oxygenation. R2: K9 Knowledge of ECMO physiology: Optimization of extracorporeal oxygenation.	4.45 (0.99) 4.65 (0.48)		5 [4–5] 5 [4–5]		93.6 96.6
R1: K10 Knowledge of ECMO physiology: Optimization of pCO2. R2: K10 Knowledge of ECMO physiology: Optimization of pCO2.	4.50 (0.68) 4.70 (0.47)		5 [4–5] 5 [4–5]		93.6 100.0
R1: K11 Knowledge of ECMO physiology: in V-A ECMO: Optimization of blood flow and native cardiac function. R2: K11a Knowledge of ECMO physiology: in VA-ECMO: Optimization of haemodynamic support, including blood flow, native cardiac function. R2: K11b Knowledge of the role of left ventricular unloading in VA-ECMO.	4.55 (0.94) 4.80 (0.41) 4.70 (0.47)		5 [4–5] 5 [5–5] 5 [4–5]		97.9 100.0 96.6
R1: K12 Knowing how to interpretation of cardiac ultrasound images during placement and follow-up. R2: K12 One member of the team should be able to interpret cardiac ultrasound images during placement, follow-up and weaning. In previous survey 'one team member' was not specified.	4.30 (0.65) 4.35 (0.98)		4 [4–5] 5 [4–5]		85.4 89.7
R1: K13 Knowledge of risks associated with the procedure. R2: K13 Knowledge of risks assessment and complications during ECMO support.	4.65 (0.48) 4.45 (0.51)		5 [4–5] 4 [4–5]		97.9 100.0
R1: K14 Knowledge of how to interpret the blood gasses, venous and arterial. R2: K14 Monitoring and interpretation of blood gas analysis during ECMO.	4.50 (0.68) 4.50 (0.76)		5 [4–5] 5 [4–5]		95.7 96.6
R1: K15 Knowledge of the weaning process of ECMO including clinical signs of pulmonary or cardiac recovery. R2: K15 Knowledge of the weaning procedure of ECMO: timing and interpretation of clinical physiological, respiratory and hemodynamic variables for both VV- and VA- ECMO.	4.70 (0.47) 4.50 (0.51)		5 [4–5] 4.50 [4–5]		95.7 100.0
R1: K16 Knowledge of ECMO weaning: pump/gas flow weaning techniques. R2: K16 Knowledge of ECMO weaning: pump flow and gas flow weaning techniques.	4.55 (0.68) 4.55 (0.51)		5 [4–5] 5 [4–5]		89.6 100.0
R1: K17 Knowledge of the principles of coagulation and anticoagulation. R2: K17 Knowledge of the principles of coagulation and anticoagulation in normal circumstances and during ECMO.	4.45 (0.60) 4.30 (0.73)		5 [4–5] 4 [4–5]		91.7 96.6
R1: K18 Being able to use and implement the local heparin protocol. R2: K18 Being able to use and implement local anticoagulation protocols.	4.45 (0.68) 4.40 (0.75)		5 [4–5] 4.50 [4–5]		89.6 96.6
R1: K19 Knowledge about outcome data of ECMO patients. R2: K19 Local outcome data of ECMO patients should be registered and discussed at least once a year.	4.00 (0.64) 4.50 (0.60)		4 [4–4] 5 [4–5]		83.0 96.4
R1: K20 Knowledge of prevention, diagnosis and treatment of recirculation. R2: K20 Knowledge of prevention, diagnosis and treatment of recirculation.	4.45 (0.60) 4.45 (0.51)		5 [4–5] 4 [4–5]		97.9 100.0
R1: K21 Knowledge of prevention, diagnosis and treatment of Harlequin syndrome. R2: K21 Knowledge of prevention, diagnosis and treatment of Harlequin syndrome.	4.75 (0.44) 4.65 (0.58)		5 [4–5] 5 [4–5]		100.0 100.0
R1: K22 Knowledge of diagnosis and policy of cardiac stunning. R2: K22 For the physician: knowledge of diagnosis, pathophysiology and treatment policy in case of cardiac stunning.	4.35 (0.93) 4.45 (0.75)		4 [4–5] 5 [4–5]		87.2 89.3
R1: K23 Knowledge of the symptoms and clinical signs of limb ischemia and prevention of it. R2: K23 Knowledge of of the symptoms and clinical signs of limb ischemia and prevention of it.	4.80 (0.41) 4.65 (0.58)		5 [4–5] 5 [4–5]		100 96.6
R1: K24 Knowledge of treatment of cardiac arrest on ECMO. R2: K24 Knowledge of treatment of cardiac arrest on ECMO.	4.50 (0.68) 4.45 (0.60)		5 [4–5] 4.50 [4–5]		95.8 96.6
R1: K25 Knowledge of the principles of lung ventilation during ECMO. R2: K25 Knowledge of the principles of mechanical ventilation during ECMO.	4.45 (0.60) 4.55 (0.51)		5 [4–5] 5 [4–5]		95.8 100.0
R1: K26 Knowledge about the influence of hemoglobin level on the required blood flow. R2: K26 Knowledge of oxygen delivery physiology including influence of hemoglobin in ECMO support.	4.25 (0.71) 4.50 (0.60)		4 [4–5] 5 [4–5]		81.3 93.1
R1: K27 Knowledge of the 'Rated Flow' is for a specific oxygenator. R2: K27 For perfusionists: knowledge of the 'Rated Flow' is for a specific oxygenator.	3.80 (0.69) 4.20 (0.69)		4 [3–5] 4 [4–5]		66.7 89.7
R1: K28 Knowledge of the indications for sedation during ECMO. R2: K28a Knowledge about sedation during ECMO. R2: K28b Knowledge of awake ECMO.	4.25 (0.63) 4.20 (0.52) 4.10 (0.44)		4 [4–5] 4 [4-4.75] 4 [4–4]		89.4 93.1 96.6
R1: K29 Knowledge of infection prevention and treatment. R2: K29 Knowledge of infections in ECMO patients: prevention and treatment.	4.20 (0.69) 4.35 (0.48)		4 [4–5] 4 [4–5]		89.6 100.0
R1: K30 Knowledge of the use of Target Dosed Monitoring of antibiotics. R2: K30 For the physician: knowledge of the use of Target Dosed Monitoring of antibiotics.	3.95 (0.75) 4.35 (0.67)		4 [3–4] 4 [4–5]		68.1 89.7
R1: K31 Knowledge of positioning and mobilization of patients on ECMO. R2: K31 Knowledge of positioning and mobilization of patients on ECMO.	4.60 (0.59) 4.40 (0.59)		5 [4–5] 4 [4–5]		89.6 96.6
R1: K32 Knowledge of the principles of ECMO nomenclature. R2: K32 Knowledge of correct ECMO nomenclature.	4.00 (0.72) 3.90 (0.64)		4 [3,5–5] 4 [3.25-4]		74.1 71.4
R1: K33 Knowledge about lung ventilation during ECMO is not recommended. R2: K33 Knowledge about lung ventilation during ECMO is not recommended.	1.45 (0.99) 1.35 (0.48)		1 [1–2] 1 [1–2]		91.0 93.0
***Technical skills***					
R1: T1 Being able to prepare and review of the checklist: i.e. ordering blood, reanimation medication, equipment. R2: T1 Every health care worker in the ECLS team should know his role.	4.35 (0.74) 4.60 (0.50)		4 [4–5] 5 [4–5]		93.6 100.0
R1: T2 Handcrancking. R2: T2 Handcrancking.	4.55 (0.68) 4.20 (0.95)		5 [4–5] 4.5 [3.25-5]		83.0 82.1
R1: T3 Be able to measure the vessel diameter and perform ultrasound guided puncture in peripheral ECMO. R2: T3 For the physician: be able to measure the vessel diameter and perform ultrasound guided puncture in peripheral ECMO.	3.70 (1.08) 4.00 (0.97)		4 [3–5] 4 [3–5]		57.4 72.4
R1: T4 Being able to insert the guidewire correct and give attention for any signs of obstruction. R2: T4 For the physician: being able to insert the guidewire correct and give attention for any signs of obstruction.	4.30 (0.86) 4.65 (0.58)		5 [3,5–5] 5 [4–5]		73.9 93.1
R1: T5 Being able to insert/connect of the cannulas correct. R2: T5 For the physician and perfusionists: being able to insert/connect of the cannulas correct.	4.25 (0.91) 4.70 (0.47)		5 [4–5] 5 [4–5]		80.9 96.4
R1: T6 Being able to prime of the circuit. R2: T6 For the perfusionists: being able to prime of the circuit.	3.60 (0.88) 4.75 (0.44)		4 [3–4,25] 5 [4.25-5]		59.6 100.0
R1: T7 Circuit checks. R2: T7 Every team member should be able to check the circuit.	4.65 (0.58) 4.50 (0.51)		5 [4–5] 4.50 [4–5]		93.6 100.0
R1: T8 Organisation of the decannulation procedure: personnel, medication, potential hazards, preparing instruments for vessel reconstruction. R2: T8 Organisation of the decannulation procedure following local protocol.	4.45 (0.60) 4.40 (0.50)		4 [4–5] 4 [4–5]		91.3 100.0
R1: T9 Transfusion of blood and blood products: why, which thresholds, how and possible complications on ECMO. R2: T9 Transfusion of blood and blood products: why, which thresholds, how and possible complications on ECMO.	4.45 (0.68) 4.35 (0.48)		5 [4–5] 4 [4–5]		89.4 100.0
R1: T10 Being able to change the oxygenator. R2: T10 For perfusionists: being able to change the circuit / oxygenator.	3.80 (1.36) 4.80 (0.41)		4 [3–5] 5 [5–5]		64.4 100.0
R1: T11 For physicians: being able to place an Avalon® cannula. R2: T11 Placement of a dual lumen canula should only be placed after multidisciplinary discussion by an experienced physician.	3.70 (0.97) 4.40 (0.75)		4 [3–4] 5 [4–5]		57.8 86.2
***Attitude***					
R1: A1 Know his/her limits and call for help if needed. R2: A1 Know his/her limits and call for help if needed.	4.75 (0.55) 4.80 (0.41)	5 [5–5] 5 [5–5]		97.9 100.0	
R1: A2 Profound situational awareness of the patient and his medical condition. R2: A2 Profound situational awareness of the patient and his medical condition.	4.45 (0.68) 4.45 (0.60)	4.5 [4–5] 4.50 [4–5]		93.6 96.6	
R1: A3 To obtain an informed consent from the patient or family. R2: A3 The physician should obtain an informed consent from the patient or family.	3.95 (0.88) 3.80 (0.89)	4 [3–4,5] 4 [3–4]		63.0 65.5	
R1: A4 Know the skills and responsibilities of the different team members. R2: A4 Know the skills and responsibilities of the different team members.	4.65 (0.48) 4.40 (0.50)	5 [4–5] 4 [4–5]		100.0 100.0	
R1: A5 For physicians: lead the team. R2: A5 There should be a team leader available on every occasion.	4.35 (0.81) 4.55 (0.60)	5 [4–5] 5 [4–5]		84.8 96.6	
R1: A6 To use closed loop communication in procedures, transport, mobilization. R2: A6 To use closed loop communication in procedures, transport, mobilization.	4.45 (0.60) 4.60 (0.50)	5 [4–5] 5 [4–5]		95.7 96.4	
R1: A7 Handovers and communication should be structured and standardized e.g., using ISBAR. R2: A7 Handovers and communication should be structured and standardized e.g., using ISBAR.	4.15 (0.81) 3.95 (0.60)	4 [3,25 − 5] 4 [4–4]		73.3 75.9	
R1: A8 There has to be an experienced team available 24/24 7/7 for troubleshooting. R2: A8 There should be an experienced team available 24/24 7/7 for troubleshooting.	4.85 (0.36) 4.75 (0.55)	5 [5–5] 5 [5–5]		100.0 96.6	
R1: A9 Be able to make difficult decisions, including when to stop the ECLS if one encounters futility. R2: A9 Difficult decisions should be made after multidisciplinary discussion.	4.55 (0.75) 4.60 (0.50)	5 [4–5] 5 [4–5]		89.1 96.6	
R1: A10 To follow-up and register patient's outcomes in a database. R2: A10 Follow-up and register patient's outcomes in a database is the task for the ECMO coordinator, data should be mandatory evaluated and benchmarked.	4.10 (0.78) 4.25 (0.44)	4 [4–5] 4 [4-4.75]		84.8 100.0	
R1: A11 Being an ELSO-member (Extracorporeal Life Support Organization). R2: A11 The centre should be registered as an ELSO-member (Extracorporeal Life Support Organization).	3.80 (0.89) 4.05 (0.68)	4 [3–5] 4 [4-4.75]		70.2 75.9	
R1: A12 Be able to consider the risks/ benefits for every ECMO run. R2: A12 Be able to consider the risks/ benefits for every ECMO run.	4.55 (0.51) 4.55 (0.51)	5 [4–5] 5 [4–5]		97.9 100.0	

## Discussion

In this International Delphi consensus on key elements for an e-learning program for ICU personnel taking care of patients treated with ECMO we identified 34 knowledge items, 10 technical skills, and 9 attitudes.

Experts, who participated in this modified Delphi questionnaire, came from Europe and Australia, with more than 10 years’ experience in a dominantly academic setting. The panel consisted of nurses, perfusionists and physicians. Our center will certainly not be the first to implement E-learning in ECMO education. In 2017, already 36% of ECMO simulation sites in the USA reported computer-based self-assessment learning modules for ECMO-practitioners.[11] The benefits could be that this is a learner centered way of teaching, resulting in an active learner in a psychological safe environment, working at his own pace, where knowledge can be tested with immediately given feedback.[19–21].

### The modified Delphi study

The modified Delphi technique has already proven its use in health care disciplines for example in developing fundamental skills, safety behavior or key interventions and quality indicators. [22–24]

This study’s response rate of 45.3% in the first round and 60.4% in the second round is comparable to Maertens et al. with a response rate of 43%. But in contrast, Hoste et al. had a response rate of 90% in a study in which only 20 experts where invited. Mostly a mix of backgrounds of professionals is used. In line with Hoste e.a., this questionnaire invited nurses and physicians.[22–24].

Internal consistency was “excellent”, as shown by the calculated Cronbach’s alfa scores. A possible explanation for this can be motivational influence, as the panelist participated voluntary and may have felt responsible to achieve important conclusions. The scores for the different competency areas (knowledge, technical skills, and attitude) decreased from the first to the second round, because of a lower number of panelists.[25] For knowledge only, the internal consistency was “good”, for technical skills it decreased from “good” to “acceptable”. For attitudes it increased, but stayed in the range of “acceptable”.[17] We also showed a good intraclass correlation with a significant interrater agreement, meaning that the experts had the same expertise or gave homogeneous answers.[14, 26].

### Fundamental knowledge skills

The top three most important knowledge skills indicate that the experts value the knowledge of ECMO physiology which is in line with existing ECMO training programs. [20, 27, 28] Recognition of limb ischemia due to cannula placement has been included in the top three of knowledge skills. This can indicate that prevention, early recognition and treatment stays important because it is peripheral placed veno-arterial ECMO care specific, more than e.g. the recognition of neurological problems or infection. Focus on recognition of limb-ischemia is, as far as we know, always included in ECMO-training programs. [28, 29]

Final consensus on 34 of 36 knowledge topics was achieved. These are topics that have been clearly described in the standard ECMO manuals registered by ELSO guidelines and in “ECMO-course for physicians and nurses” of the Leiden University Medical Center, comprising basic pathophysiology, mechanical-human interaction and problem-solving for life-threatening situations.[8, 19] In contrast, the following topics were not included in our knowledge topics: “the history of ECMO”, neither specific neonatal or pediatric topics, nor congenital heart pathologies. In contrast, knowledge of the different prediction scores was not retained as core competency. This may be explained by the fact that scores seldom directly affect daily practice of ECMO-patients. Although these topics were not withheld in another paramedic and registrar training, they are mentioned as a possible predictive tool in “the ELSO Red Book”. Moreover, correct indications may optimize the outcome scores and will increase the value of ECMO as a recommendable salvage therapy; correct tariffication and related data registries should evaluate quality-based programs and justify proper financing.[30, 31].

### Fundamental technical skills

The consensus in the first round regarding the proficiency of technical skills was low 54% but rose to 90% in the second round. The adaptation of the statements has led to a significantly higher consensus among the experts, 10/11 were in fact retained as essential. The panelists gave the following statements the highest scores: “For perfusionist; being able to change the circuit/oxygenator.“, “For the perfusionist: being able to prime the circuit.“, “For the physician and perfusionist; being able to insert/connect the cannulas correctly.“ Apparently, all respondents value the dedicated service of the perfusion department in supporting device maintenance and set-up, managing technical problems, implementing safety checks, and optimizing best practice. Statements on technical aspects were thoroughly commented and were modified and shifted in emphasis consequently. The selected technical skills are in line with technical trainings described by other authors and so they are brought together and confirmed by our modified Delphi.[11, 28, 32, 33] This study adds that the different responsibilities and tasks within these technical operations become more explicit in contrast to the position paper of the ECMO-net, that compiles different technical skills and puts emphasis on working in multidisciplinary teams. [4] Raffelli e.a. follow a “dual provider” model, wherein specific tasks are dedicated to specific professional groups, but these specific technical skills are not listed for each profession. [20]

### Fundamental behavioral skills

Consensus was reached in 75% of the statements for both rounds: nine attitudes were scored essential to team members in daily ECMOcare. Emphasis was put on emergency situations, safety principles and weighing the risk-benefit balance, with top ranking for: “learn to assess your own knowledge and skills correctly” and “call for help when necessary”. The consensus also highlights the need to have a well-trained ECMO team available 24/7. The fact that the expertise can only increase when there is sufficient exposure, emphasizes that this supportive treatment should be offered in specialized centers.[1, 34] Although e-learning may be more learner-oriented, the e-learning should emphasize the importance of the team approach, in which each individual specializes in his professional actions and behaviors. The behavioral skill “to ask for an informed consent”, which also is proposed by the ELSO guidelines for training ECMO-specialists, did not reach consensus of the experts, but in contrast all other attitudes defined as essential are not part of the ELSO guidelines and were proposed by the research group based on the experience of the Ghent University Hospital ECMO simulation team.

The results of this Delphi Consensus are in parallel with key aspects of good practice presented by the International ECMO network and the ELSO.[4, 19, 20] The ELSO guidelines grew upon expertise in teaching HCP trough the last decades and educational programs were developed on expert opinion. This guidelines date from 2010 and served well in developing this Delphi questionnaire.

### Differences in scoring depending on educational background

In the first round, twelve statements were retained with a significantly different grading between the experts with different background, but in the second round, these differences were no longer significantly different. This may be explained by rephrasing the statements and sharing the scores obtained in the first round allowing experts to reach consensus Additionally, the lower response rate in the second round with fewer perfusionists may have influenced this result. There were 32 vs. 21 physicians participating, 4 vs. 3 nurses and 12 vs. 5 perfusionists participating in the first and second rounds, respectively.

### Limitations

We want to discuss the following limitations to this study. Only 60.4% (29 of 48) of the initial participants, responded in the second round, possibly leading to selection bias. The dropouts possibly can be explained by a global surge in the COVID 19-pandemia, which occurred when the experts were asked to fill out the second round.[3, 35] At the start of this survey 106 experts were contacted by an automatic generated e-mail by the software RedCap®. Given the initial low response rate in the first round, we contacted some of the invited colleagues, who indicated not have been contacted by e-mail from RedCap®. RedCap® technical support clarified that the generated e-mails may have been blocked by hospital firewalls. Another limitation of the study is that no experts from the USA, Latin America or Asia participated.

Experts in the US were contacted for the first round, none participated in the Delphi questionnaire, one did send an email that he liked the design but was too busy with a considerable amount of clinical work.

Also important to mention is the low rate in nurses and perfusionists that collaborated in the Delphi questionnaire. Seven out of ten experts were physicians in the second round.

The number of questions may also have deterred experts from participating.

## Conclusion

Although ECMO is frequently used to support patients in ICU, the process needed to determine essential educational topics has not been published. By establishing this modified Delphi consensus, an expert opinion was achieved about the content of “ECMO essential skills” among physicians, ICU nurses and perfusionists anno 2021. This consensus was mainly created with participation of physicians, as the response rate for nurses and perfusion decreased in the second round. In the topics knowledge, technical skills, and attitudes essential skills were identified by experts and are now used to guide the development of an e-learning module. These essential skills will also enhance simulation scenarios in the hands-on training sessions. Whether this e-learning and simulation-based training will provide better care, is the subject for a subsequent study.

## Electronic supplementary material

Below is the link to the electronic supplementary material.

Supplementary Material 1

## Data Availability

The datasets from this modified Delphi questionnaire analyzed during this study are available from the corresponding author on reasonable request.

## Abbreviations

ECMO: Extra Corporeal Membrane Oxygenation.
ELSO: Extracorporeal Life Support Organization.
HCP: health care professional.
ICC: intraclass correlation coefficient.
ICU: intensive care unit.
RedCap®: Research Electronic Data Capture®.
VA-ECMO: Venoarterial ECMO.
VV-ECMO: Venovenous ECMO.
