# Supplementary material for: Adult essential extracorporeal membrane oxygenation (ECMO) skills for use in an e-learning program for ICU physicians, nurses and perfusionists: a consensus by a modified Delphi questionnaire
Source: BMC Med Educ. 2022 Nov 14;22:786. doi: 10.1186/s12909-022-03764-2 (PMC9662121; doi:10.1186/s12909-022-03764-2)
Supplement: Supplementary file 1 — Supplementary Material 1 [file 12909_2022_3764_MOESM1_ESM.docx]

Supplement

Statistically significant differences in ratings between the first and second Delphi questionnaire, were found for eight statements. These eight statements on skills were adjusted between the two rounds on advice of the experts to achieve greater consensus. (Table S1)

Table S1. Significant changes in ratings between the two Delphi rounds

| Skill | Rating round 1  median [IQR] | Rating round 2  Median [IQR] | Consensus  % | p |
| --- | --- | --- | --- | --- |
| R1: When to convert to another ECMO construction.  R2: For the physician and perfusion: when to change the ECMO configuration.” | 4.0 [4.0;5.0] | 5.0 [4.0;5.0] | 89.66  100 | 0.001 |
| R1: When to convert to another ECMO construction.  R2: For physician and perfusionist: how to change the ECMO configuration. | 4.0 [4.0;5.0] | 5.0 [4.0;5.0] | 89.66  100 | 0.012 |
| R1: Influence of hemoglobin level on the required blood flow.  R2: Oxygen delivery physiology including influence of hemoglobin in ECMO support. | 4.0 [4.0;5.0] | 5.0 [4.0;5.0] | 79.31  93.10 | 0.002 |
| R1: Knowing what a ‘Rated Flow’ is for a specific oxygenator.  R2: For perfusionists, knowing what a ‘Rated Flow’ is for the specific oxygenator. | 4.0 [3.0;5.0] | 4.0 [4.0;5.0] | 62.07  89.66 | 0.015 |
| R1: The use of Target Dosed Monitoring of antibiotics.  R2: For the physician, the use of Target Dosed Monitoring of antibiotics. | 4.0 [3.0;4.0] | 4.0 [4.0;5.0] | 60.71  89.66 | 0.027 |
| R1: Priming of the circuit.  R2: For the perfusionists: priming of the circuit. | 4.0 [3.0;4.0] | 5.0 [5.0;5.0] | 100 | <0.001 |
| R1: Being able to change the oxygenator.  R2: For the perfusionists: being able to change the circuit / oxygenator. | 4.0 [2.0;5.0] | 5.0 [5.0;5.0] | 66.67  100 | 0.001 |
| R1: For physicians: being able to place an Avalon cannula.  R2: Placement of a dual lumen canula should only be done after multidisciplinary discussion by an experienced physician. | 4.0 [3.0;4.0] | 4.5 [4.0;5.0] | 66.67  86.21 | 0.018 |

R1: Round 1

R2: Round 2

Consensus in this study was defined by 80% of experts that rates a skill with a score of 4 or more.
